# Framework for policymaking on self-management of health by older adults using technologies

**DOI:** 10.1186/s12961-024-01119-5

**Published:** 2024-03-05

**Authors:** Amélie Gauthier-Beaupré, Craig Kuziemsky, Bruno J. Battistini, Jeffrey W. Jutai

**Affiliations:** 1 Faculty of Health Sciences and Life Research Institute, University of Ottawa, Ottawa, ON Canada; 2 School of Business, MacEwan University, Edmonton, AB Canada; 3 Interdisciplinary School of Health Sciences, University of Ottawa, Ottawa, ON Canada

**Keywords:** Policymaking, Information and communication technology, Chronic disease self-management

## Abstract

**Background:**

During the coronavirus disease 2019 (COVID-19) pandemic, the use of information and communication technologies (ICTs) to support care management exponentially increased. Governments around the world adapted existing programs to meet the needs of patients. The reactivity of governments, however, led to changes that were inequitable, undermining groups such as older adults living with chronic diseases and disability. Policies that align with recent developments in ICTs can promote better health outcomes and innovation in care management. A framework for policymaking presents potential for overcoming barriers and gaps that exist in current policies.

**Objective:**

The goal of this study was to examine how well a provisional framework for policymaking represented the interactions between various components of government policymaking on older adults’ self-management of chronic disease and disability using ICTs.

**Methods:**

Through an online survey, the study engaged policymakers from various ministries of the government of Ontario in the evaluation and revision of the framework. The data were analyzed using simple statistics and by interpreting written comments.

**Results:**

Nine participants from three ministries in the government of Ontario responded to the questionnaire. Overall, participants described the framework as useful and identified areas for improvement and further clarification. A revised version of the framework is presented.

**Conclusions:**

Through the revision exercise, our study confirmed the relevance and usefulness for a policymaking framework on the self-management of disease and disability of older adults’ using ICTs. Further inquiries should examine the application of the framework to jurisdictions other than Ontario considering the dissociated nature of Canadian provincial healthcare systems.

**Supplementary Information:**

The online version contains supplementary material available at 10.1186/s12961-024-01119-5.

## Background

Government policies must modernize to account for changing needs, new research and emerging discoveries. Through effective knowledge translation, policymakers can more easily apply and implement new knowledge [1, 2]; however, policymakers still encounter difficulties integrating new knowledge into policies since innovation and research occur at such a fast pace [3]. Digital health, which includes digital technologies such as medical connected tools that aim to improve health [4], is a good example of something that is not well represented in policy systems. Technologies, such as information and communication technologies (ICTs), are changing the way individuals care for and manage their chronic diseases and disabilities and promote increased opportunities for self-management. Research evidence point to the benefits of using ICTs to self-manage health for diverse groups of individuals, including older adults. The evidence suggests benefits to factors such as health outcomes and empowerment [5, 6] and effective care management and promotion of healthy lifestyles [7–10]. Effective integration of ICTs within the lives of individuals and patients who can self-manage their conditions relies heavily on policies and services that support their equitable access and use [11]. The COVID-19 pandemic provided a concrete example of the lag, which already existed, between innovations and their integration into policies. Prior to the COVID-19 pandemic, there had been limited efforts from organizations to include digital options for care delivery and health services even if ICTs already existed and were proven to be effective [12]. The pandemic led to reactive policymaking related to services and programs, where in most cases, there was a shift to digital modes of care delivery for people with chronic diseases and disabilities to meet public health requirements such as physical distancing [13, 14]. This reactive policymaking led to mixed outcomes for several groups of the population, and even sometimes growing the digital divide leading to inequities in access to healthcare [15]. For certain older adults in the province of Ontario in Canada, digital technology facilitated the continuation of care routines and ensured safety, while for others, it exacerbated experiences of social isolation and negatively impacted care management [16].

Proactively developing timely policies that consider the role of ICTs in supporting care could lead to better and more equitable programming and services over time. As such, policies can serve as a lever for organizations to consider innovative care management strategies.

### Prior work

**Fig. 1:**
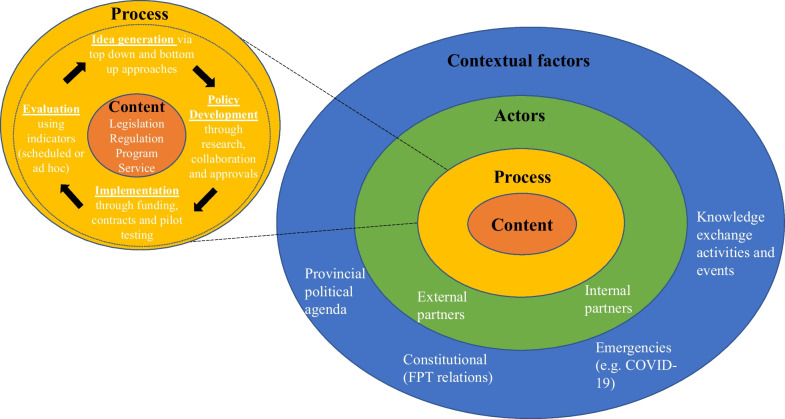
Framework for policymaking on older adults’ SMDD using ICTs

Governments around the world are promoting self-care initiatives, but research that supports policymaking on the issue is lacking [17]. In Canada specifically, there has been limited research on policymaking for older adults’ self-management of disease and disability (SMDD) using ICTs. An examination of policies on self-management of chronic diseases reported in 2014 illustrated the divergence of policies on the topic across the country [18]. For Ontario specifically, much of the policy work on self-management was for disease-specific programming (mainly for diabetes) [18], with limited policies focussing on technology as an enabler of self-management [19]. In addition, there are several policy barriers that need to be addressed by policymakers to enhance implementation of digital health technologies. These include (i) the need to clarify definitions of digital health innovation, (ii) be able to articulate a clear mission for transformation and (iii) clarify processes and use change management approaches to policymaking [20]. To support such an endeavour, we engaged policymakers of various ministries who are responsible for older adults’ policies in a study to discover and map the policymaking environment in Ontario on the topic of older adults’ SMDD using ICTs [16]. Key components that make-up the policymaking environment were identified and presented in a framework (Fig. 1) [16]. The provisional framework (Fig. 1) include factors of context (provincial political agenda, constitutional FPT relations, emergencies and knowledge exchange activities and events), actors (external partners and internal partners), process (idea generation, policy development, implementation and evaluation) and content (legislation, regulation, program and service) [16]. The intention behind the creation of this framework was to introduce the intersections of the different components of policymaking, the influential role that each factor may play on others and to highlight the complexity associated with policymaking on the topic of older adults’ SMDD using ICTs [16].

The complex system of factors that influence policymaking (Fig. 1) requires a means of understanding the interactions between them to account for unintended consequences (i.e. issues of inequity and digital divide). Frameworks are high-level descriptions that are applicable to a wide range of scenarios. They employ concepts and their interrelationships to offer a frame of reference within which to organize and focus decision-making and assist interpretation of research evidence and other contextual information [21, 22]. There are several conceptual frameworks for policymaking [23–25] and care management [26–28], but no evidence was identified of their application to older adults’ SMDD using ICTs. Policies present as a tool to identify priorities that need to be integrated into practice, and currently, there exists a significant gap and lack of connection between policy and practice. For this reason, we build upon on earlier evidence-based research [16] to further develop and refine a realistic framework for policymaking on older adults’ SMDD using ICTs.

### Goal of this study

This present study’s main objective was to address the following research question: How well does the framework recently proposed by Gauthier-Beaupré et al. [16] capture the complex set of interactions involved in government policymaking relative to the topic of older adults’ SMDD using ICTs? We conducted a survey of policymakers currently working within the government of the province of Ontario, Canada, to revise and refine the framework.

This study aimed to address the following specific objectives:

1. Examine the clarity, understandability and completeness of a provisional framework for policymaking on the topic older adults’ SMDD using ICTs;
2. Assess the applicability and usefulness of the proposed provisional framework for policymaking on the topic of older adults’ SMDD using ICTs within an existing policymaking environment; and
3. Refine the provisional framework to enhance its applicability, usefulness and sustainability within a policymaking environment specifically focussed on older adults’ SMDD using ICTs.

## Methods

### Ethics approval

This study was approved by the University of Ottawa’s Health Sciences and Sciences Research Ethics Board (ethics file no. H-07-20-5555). Consent to participate in this study was obtained implicitly through the survey. An implied consent form was attached to the invitation emails and contained all details about the study, benefits, risks, confidentiality and anonymity, conservation of data, voluntary participation and information about the study results. By clicking on the survey link, participants gave their implicit consent to participate in the study.

### Study design

This study used a descriptive quantitative approach supplemented by qualitative comments to assess and refine the provisional framework for policymaking on older adult’s SMDD using ICTs. A survey method (cross-sectional) was selected to effectively assess different components of the framework and allowed for participants to include written comments to explain their views and responses as needed.

### Participants and recruitment

Participant selection was performed using criterion sampling, which is based on predetermined criteria of importance for selecting participants [29]. This project recruited policymakers within specific sectors the government of Ontario that could provide relevant insights regarding the provisional framework. Participants were identified and selected on the basis of the ministry in which they were employed. The selection was limited to those employed in the Ministry of Health, the Ministry for Seniors and Accessibility, the Ministry of Children, Community and Social Services and the Ministry of Economic Development, Job Creation and Trade. The sample size was determined according to the number of participants required to obtain data saturation. To determine data saturation, we relied on the repetition of previous participant accounts, which indicated that any further data collection would not yield different experiences [30]. We aimed to obtain at least one individual working in each of the targeted ministries of the government of Ontario to obtain a diversity of perspectives. Once the ministries were identified, we obtained the contact information of potential participants via the online government of Ontario Employee and Organization Directory (INFO-GO). Participants were eligible to participate if they currently worked in the identified ministries of the government of Ontario and were potential users of the framework as a result of the nature of their work.

Invitation emails were sent to 54 potential participants between 18 July 2022, and 20 September 2022. In the first round of invitations, we targeted policymakers who had previously participated in earlier stages of consultations and who would be considered potential users of the framework. Follow-up emails were sent twice during a 2-week interval. We also used snowball sampling, in which existing participants referred other potential participants to the researchers.

### Survey development and administration

Data were collected using an online survey through the survey platform SurveyMonkey (San Mateo, California, USA; www.surveymonkey.com). The survey was organized into four sections: (A) demographics, (B) the concept map – form and content, (C) setting – people and organizations as implementers and (D) additional thoughts and comments. These sections were selected and derived from the behavioural theory in implementation, which indicates that implementation performance of policies rely on three clusters of independent variables: (A) form and content of the framework, (B) capacity of organizations responsible for the program and (C) qualifications of people in charge of operations [31]. The survey (Additional file 1) required participants to analyze, comment and propose modifications and additions to the provisional framework for policymaking (Fig. 1). To gather perspectives on the form and content of the framework, the survey questions focussed on assessing the framework’s usefulness and the importance of concepts it presented. Questions revolved around the understandability, completeness, appearance and applicability of the framework. To assess the importance of the concepts presented, we asked questions that focussed on the degree to which each concept of the framework was viewed as impactful to policymaking. To assess the organizational factors impacting the framework’s use and operationality, the survey questions were developed to assess implementation barriers and facilitators.

In the survey, we used the term ‘concept map’ to define the visual representation of the framework and to allow participants to better understand how the framework was constituted of in preliminary phases of development. When inquiring about broader representation and applicability of the concept map, the survey used the term ‘framework’.

### Data management and analysis

This study used a mixed methods approach to analyze and combine both quantitative and qualitative data. Quantitative data from the survey were entered into SPSS, summarized using simple descriptive statistics and reported as per the guidelines for reporting descriptive statistics [32]. Written comments were analyzed using thematic analysis for each individual question and then integrated with the quantitative results. Additionally, we grouped data by the type of assessment they provided including evaluative ratings, usefulness ratings, definitional clarity ratings and importance ratings. Evaluative ratings were used to gather the perceptions of participants in regards to different components of the framework, usefulness ratings focussed on the applicability of the framework within existing policymaking settings, definitional clarity ratings sought participants’ insights on the clarity of the pre-defined definitions used to describe the concepts of the framework and importance ratings sought to identify the level of importance attributable to each of the concepts within the framework. Other open-ended comments were analyzed separately.

## Results

### Demographics

Table 1 presents a summary of participant demographics. The study’s nine participants represent diversified expertise and experiences across three different ministries of the government of Ontario. The participants represented a diverse workforce with positions ranging from advisors to directors. These positions represent the managerial level of policymakers coordinating the files and developing the policies and programs related to older adults’ SMDD using ICTs. Participants were specifically selected at this level for their strategic comprehension of policymaking. While most participants had been working in the government of Ontario for 5 years or more, all except one participant had been in their current roles for 5 years or less.

**Table 1:** Participant characteristics (*N* = 9)

Participant identifier	Ministry	Position	Years in current position	Years in Ontario Government
P1	Ministry for Seniors and Accessibility	Manager	1–2 years	5–10 years
P2	Ministry for Seniors and Accessibility	Director	2–5 years	11–20 years
P3	Ministry of Health	Manager	5+ years	11–20 years
P4	Ministry for Seniors and Accessibility	Program Advisor	2–5 years	> 20 years
P5	Ministry for Seniors and Accessibility	Team Lead	2–5 years	5–10 years
P6	Ministry of Health	Director	2–5 years	5–10 years
P7	Ministry of Economic Development, Job Creation and Trade	Director	2–5 years	11–20 years
P8	Ministry of Economic Development, Job Creation and Trade	Senior Sector Advisor	1–2 years	5–10 years
P9	Ministry of Economic Development, Job Creation and Trade	Program Manager	2–5 years	> 20 years

Of the nine participants, eight fully completed the survey and one only answered some of the questions. This aligns with the study protocol that indicated that participants could skip any questions of the survey.

### Close-ended assessment of the framework

The tabulated responses to the survey questions and associated comments from the respondents are presented in Tables 2, 3.

**Table 2:** Evaluative ratings

Level of agreement with statements	Strongly agree	Agree	Somewhat agree	Neither agree nor disagree	Somewhat disagree	Disagree	Strongly disagree
	*n*	*n*	*n*	*n*	*n*	*n*	*n*
The concept map describes well the current and overall policymaking process used by our group	0	5	3	0	1	0	0
The concept map is visually appealing	0	4	3	0	0	0	1
The format makes it easy to understand	0	5	2	0	1	0	0
The concept map is usable in its present form	0	2	2	1	3	0	0
I have the necessary expertise to understand such a concept map in my policymaking activities	1	6	0	0	1	0	0
I have the necessary expertise to implement such a concept map in my policymaking activities	2	2	2	0	2	0	0
I view this concept map as sustainable over time	0	3	4	1	0	0	0
This concept map enables a continuous monitoring and integration of innovation in policymaking	0	3	2	3	0	0	0
I see the value of a framework to guide policymaking	1	4	3	0	0	0	0
The Government of Ontario and my organization could benefit from a framework to guide policymaking	1	3	2	2	0	0	0
The Government of Ontario and my organization are ready to implement a framework such as the one proposed	0	2	5	0	1	0	0
The Government of Ontario and my organization have the necessary human and financial resources to implement this framework	0	0	3	4	0	1	0
Total	5	39	31	11	9	1	1

*N* = 8 for all statements except for ‘The concept map describes well the current and overall policymaking process used by our group’ (*N* = 9)

#### Evaluative ratings

The evaluative ratings (Table 2) reveal high levels of agreement with the statements about the framework and with the organizational aspects related to the framework. Participants’ ratings offer insights about the form and content of the framework, and the internal capacity of the Government of Ontario to implement the framework, which are presented below.

##### Form and content of the framework

Participants gave positive ratings to the visual appearance and clarity of the framework. In certain comments (Table 3), participants also provided constructive feedback to improve the framework’s visual appearance and clarity. Overall, the findings suggest that the concepts of the framework were appropriately represented and aligned with current methods for engaging in policymaking. Regarding suggestions for improving the visual appearance, two participants noted that ‘the elements work well but would benefit from a professional graphic designer. People like stuff that looks cool’ (P7) and that ‘once all of the elements are in place, it would be worth investing some time to make it visually appealing’ (P1).

**Table 3:** Comments associated with evaluative ratings

Level of agreement with statements	Comments (quotes from participants)
The concept map describes well the current and overall policymaking process used by our group	It’s a bit simplistic. In practice a wider range of contextual factors come into play including current state practices and capabilities. The descriptions of actors and processes are also highly generic Under the AODA [Accessibility for Ontarians with Disabilities Act] we are legally required to integrate internal/external partners in the idea generation phase The actors include more than internal and external partners. They also include stakeholders and citizens (who aren’t necessarily part of a partnership group)
The concept map is visually appealing. The format makes it easy to understand	The map would benefit from professional graphic design. The concepts are good The image is not visually appealing, and it’s not immediately clear where to start
The concept map is usable in its present form	The elements work well but would benefit from a professional graphic designer. People like stuff that looks cool. This is not a criticism but professional designers can add weight through visual appeal As noted previously, it’s quite generic and misses some key elements of what tends to inform real-world decision-making

The usefulness of the framework had equal positive and negative ratings, which indicates certain limitations in its applicability to existing policy environments. One participant (P6) suggested that the framework did not fully represent the policymaking process and may be missing some key elements. On the contrary, however, P8 stated that ‘th[e] map [was] very comprehensive’. This mixed rating denotes some level of uncertainty with the level of detail provided in the framework.

##### Internal implementation capacity

The assessment of the internal capacity of the government to implement this framework focussed on two domains: human resource expertise and organizational readiness. For human resources, participants positively rated their ability to understand and implement the framework. However, organizational capacity with regards to human and financial resources were given more neutral ratings, which indicates that there may be some organizational limits to implementing the framework.

#### Usefulness ratings

To better understand the usefulness of the framework, participants were asked questions about its ability to support key policy activities. Participants gave a positive rating of the framework for its ability to help engage in key domains of policymaking such as identifying the problem or issue, identifying appropriate policy solutions, identifying and describing policy options and developing a strategy for furthering adoption of a policy solution (Table 4). However, the two other policymaking activities, that is, assessing policy options and prioritizing policy options, received mixed ratings from participants. These two activities could have been viewed as different than the others since they generally require more complex processes which are not represented in this framework. As was pointed out by participants, some key elements of real-world decision-making were missing, and certain stakeholders were not included in the framework. This may also have impacted ratings of usefulness for these two activities. For example, it is likely that human and other resources not specified in the framework would be needed in assessing and prioritizing policy options.

**Table 4:** Usefulness ratings

Would this concept map help you/your group do the following?	Yes	No
	*n*	*n*
Identify the problem or issue	8	1
Identify an appropriate policy solution	7	2
Identify and describe policy options	7	1
Assess policy options	5	4
Prioritize policy options	5	3
Develop a strategy for furthering adoption of a policy solution	7	1

*N* = 9 for all options except for ‘Identify and describe policy options’ (*N* = 8), ‘Prioritize policy options’ (*N* = 8) and ‘Develop a strategy for furthering adoption of a policy solution’ (*N* = 8)

#### Definitional clarity ratings

The framework presented to participants was supplemented with definitions about each of the concepts. In the survey, participants were asked to rate and propose modifications to those definitions. Overall, participants indicated that all definitions contained a certain level of clarity, being mostly very clear, with some requiring minor modifications and few requiring major modifications (Table 5).

**Table 5:** Ratings of definitional clarity

Clarity of definitions	Very clear	Minor modifications needed	Major modifications needed	Not clear at all
	*n*	*n*	*n*	*n*
*Context*	3	3	2	0
Provincial political agenda	5	3	0	0
Constitutional federal, provincial and territorial (FPT) relations	2	6	0	0
Emergencies (e.g. COVID-19)	7	1	0	0
Knowledge exchange activities and events	7	1	0	0
Total	24	14	2	0
*Actors*	4	4	0	0
External partners	4	4	0	0
Internal partners	6	2	0	0
Total	14	10	0	0
*Process*	6	1	1	0
Idea generation	4	4	0	0
Policy development	6	2	0	0
Implementation	4	3	1	0
Evaluation	5	3	0	0
Total	25	13	2	0
*Content*	5	3	0	0
Policy	6	1	1	0
Programs	5	3	0	0
Service	4	3	1	0
Total	20	10	2	0
Overall total	83	47	6	0

*N* = 8 for all definitions assessed

##### Context

For context factors of the framework, participants had mixed opinions with regard to the clarity of the definition. The assessments for clarity ranged between being very clear, requiring minor modifications and requiring major modifications. Within the subcategories of context, answers also differed. For the provincial political agenda, participants noted that the definition was mostly very clear. For constitutional federal, provincial and territorial (FPT) relations, participants mostly indicated that the definition required minor modifications. For emergencies (e.g. COVID-19) and knowledge exchange activities and events, participants had consistent responses indicating that the definition was very clear.

##### Actors

For actor components of the framework, participants were split, where 50% (*n* = 4) indicated that the definitions for actors and external partners were very clear and 50% (*n* = 4) indicated that they required minor modifications. For the most part (*n* = 6), participants indicated that the definition for internal partners was very clear.

##### Process

For process, most of the participants were very clear about the definition provided. For the subcategories of idea generation and implementation, responses were mixed between very clear and needing a minor modification. For the subcategories of policy development and evaluation, participants mostly reported that the definitions were very clear, with a few (*n* = 2 and *n* = 3, respectively) indicating that the definitions needed minor modifications.

##### Content

For the content factor of the framework, participants reported that the definition was mostly clear, with a few (*n* = 3) indicating the need for minor modifications. For the subcategory of policy, participants rated it as being mostly very clear with the definition provided. However, the definitions provided for the programs and services received mixed ratings, where some reported the definitions were very clear (*n* = 5 and *n* = 4, respectively) and others reported that minor modifications were necessary (*n* = 3 for both).

Overall, the findings for the assessment of definitional clarity indicate that the definitions accurately represent the concepts, but some clarifications may be necessary for some.

#### Importance ratings

Participants were also asked to determine the level of importance for each of the concepts presented in the framework (Table 6). The participant ratings indicated that most concepts presented were very or extremely important and some at least moderately important for policymaking on the topic of older adults’ SMDD using ICTs. The political agenda surpassed all other concepts as being extremely important. Second, the results indicate that participants viewed content of policies and services as extremely important and very important. The least important concept in the views of participants was the concept of knowledge exchange activities and events.

**Table 6:** Ratings of importance of key concepts

Level of Importance	Extremely	Very	Moderate	Neutral	Slight	Low	Not at all
	*n*	*n*	*n*	*n*	*n*	*n*	*n*
*Context*	4	4	0	0	0	0	0
Provincial political agenda	6	1	1	0	0	0	0
Constitutional FPT relations	2	1	4	1	0	0	0
Emergencies	2	5	1	0	0	0	0
Knowledge exchange activities and events	1	1	5	1	0	0	0
Total	15	12	11	2	0	0	0
*Actors*	3	3	0	0	0	0	0
External partners	4	2	2	0	0	0	0
Internal partners	3	2	2	1	0	0	0
Total	10	7	4	1	0	0	0
*Process*	3	5	0	0	0	0	0
Idea generation	2	4	2	0	0	0	0
Policy development	4	1	3	0	0	0	0
Implementation	4	4	0	0	0	0	0
Evaluation	1	5	1	1	0	0	0
Total	14	19	6	1	0	0	0
*Content*	5	3	0	0	0	0	0
Policy	4	4	0	0	0	0	0
Programs	4	4	0	0	0	0	0
Service	5	3	0	0	0	0	0
Total	18	14	0	0	0	0	0
Overall total	57	52	21	4	0	0	0

*N* = 8 for all options except for *Actors* (*N* = 6)

### Open-ended assessment of the framework

The survey included several open-ended questions to capture information that may have been omitted from the framework and not asked in the survey. Specifically, the survey asked participants to list additional factors and concepts that they believed were important for policymaking on older adults’ SMDD using ICTs. Participants identified and listed three main points: (1) the role of companies, (2) capabilities of the state to follow innovations in technology and clinical practice and (3) stakeholder engagement. The role of companies was said to be critical to include in a framework focussed on policymaking involving technology and innovation. As mentioned by P7, there needs to be ‘a greater emphasis on the role of companies’. Companies may already be included or embedded within various concepts of the framework, ‘but the forces and innovation coming from service and technology advisors may deserve a more specific call out’ (P7). Similarly, another participant emphasized the importance of state capability within a society that ‘innovate[es] in the technology market or clinical practice’ (P6) coupled with “capacity of service delivery partners’ (P6). As such, participants stressed the necessity to consider other players in the policymaking sphere. P3 supports this claim by indicating the need for ‘stakeholder engagement through the process cycle’.

The survey also asked participants to identify strategies to integrate innovations in technology within the government policies, programs and services. Participants listed several key factors to consider, including the role of technology and its value, diversity of stakeholders consulted during policymaking and the engagement process. First, the role of technology in SMDD needs to be better understood by policymakers to allow them to effectively integrate them within their policies. Specifically, participants mentioned the need for ‘better recognition that technology and services it drives is central to the “persons” self-management’ (P7) and that governments should have ‘improved understanding of the value of ICT’ (P1). Second, participants denoted several groups of stakeholders that need to be involved in the integration of innovations within policies. For example, many participants discussed the role of companies and innovators as key and valuable stakeholders that cannot be ignored in policymaking. Specifically, P7 mentioned that governments should ‘challenge companies and innovators to solve problems through revised incentives’. Participants also believed that there was a role for ‘advocacy groups’ (P3) as well as other groups and organizations such as ‘persons with lived experience, community members, [other levels of] government (FPT), public health units’ (P1). Third, participants believed that engagements should be at the forefront for ensuring better integration of innovations in technology within policies, programs, and services. Engagement needs occur at multiple levels and with diverse individuals to ‘determine gaps’ (P1). Participants mentioned that engagement should occur with ‘technology and services innovators’ (P7) and ‘users, patients and seniors’ (P3). P3 even noted that ‘engagement of seniors/users is critical’. This level of engagement would ensure that all policy solutions follow a ‘user-friendly and user-centred’ (P3) approach.

In addition, with the understanding that policies are developed at a specific timepoint but live within a world that evolves, the survey asked participants their thoughts on how to develop sustainable policies on the topic of older adults’ SMDD using ICTs. Participants identified three main areas that they believed would ensure sustainable policymaking: (1) commitment and investment, (2) supportive systems and expert professionals and (3) collaboration and cooperation. First, participants identified commitment and investment from the government as key features to ensure policies are sustainable. Participants noted that this commitment could be done by ‘prioritization’ (P3) in which policymakers make decisions on the basis of the sorting of priorities, technologies to support older adult’s SMDD is a ‘continued priority’ (P3) and there is a constant ‘evaluat[ion] [of] new opportunities’ (P7). While it was suggested that there be ‘resource investments [and] long-term commitment[s]’ (P8), participants shared that policymakers need to be ‘ab[le] to measure budget outlays against deferral of future/downstream system costs’ (P7). As was pointed out by P6, ‘it is difficult to tie outcomes (e.g. improvements in quality of life or health system resource utilization) to investments made in ICTs. If these linkages could be better made then it would allow for more stable and predictable support from government’. Second, participants discussed the importance of a supportive system which incorporates learning and development opportunities at all levels (policymakers and stakeholders). Participants revealed that the creation of sustainable policies would only be possible with ‘deeper training of policy professionals to understand technology’ (P7) and with ‘improved data analysis capabilities within government’ (P1). In alignment with that, P7 also mentioned the need for ‘patients/citizen […] to be trained at many levels to manage their care’. Beyond creating these learning opportunities, participants noted that a supportive system would also consider ‘privacy laws that allow for flexibility and ensures privacy’ (P1). Third, participants revealed that sustainability cannot exist without collaboration and cooperation. The ‘ability to share data across government ministries for policymaking’ (P1) and ‘broader co-operation and commitment to delivering the product’ (P8), were noted as enabling sustainable policymaking.

Overall, open-ended assessments of the framework provided detailed information about missing components in the framework, ways to integrate innovation into government policies and defined key enablers to sustainable policymaking. With regard to additions to the framework, participants mentioned the need to include companies as a distinct system, include capabilities of governments to keep up to speed with innovations and the role of stakeholder engagement throughout the cycle. For integration of innovations, participants noted the need for policymakers to value the role of technologies, consult a diversity of stakeholders in their policymaking activities and the need for engagement at multiple levels of policymaking. Finally, to ensure sustainability of policies, participants noted that it was mandatory to obtain commitment and have investments in the sector, have supportive environments with experts on the topic and allow for collaborative and cooperative processes.

### Summary of the findings

The results point to an overall positive rating of the provisional framework’s clarity, understandability and completeness for policymaking on the topic older adults’ SMDD using ICTs. Both the clarity and the understandability of the framework were viewed positively by participants since there was a general perception that it presented a good level of detail and had a good visual appearance. Participants also offered some recommendations to strengthen the framework’s completeness such as adding additional stakeholders and sectors to the overall landscape.

The applicability and usefulness of the provisional framework for policymaking on the topic of older adults’ SMDD using ICTs was also positively perceived by participants. There was a general sense that the framework was useful because it aligned with current ways of engaging in policymaking and that it clearly represented the collaboration that occurs within the policy environment. As such, the framework would be applicable to the existing policymaking environment. However, the capacity of organizations to implement the framework within the existing structure was deemed to be limited by organizational readiness due to factors such as financial resources, even if the personnel implementing the framework would have the necessary expertise to do so.

## Discussion

### Principal findings

**Fig. 2:**
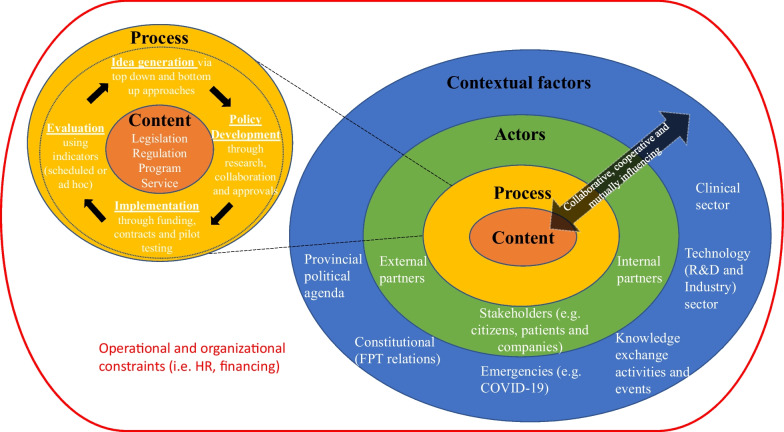
Changes to the framework for policymaking on older adults’ SMDD using ICTs

The results suggest that the provisional framework for policymaking on older adults’ SMDD using ICTs (Fig. 1) adequately captured the basic components that should compose the framework. The visual representation and comprehensiveness of the framework were noted as areas for improvement. While the definitions of concepts of the framework were considered mostly adequate, the results suggest that they could be improved in all four areas: context, actors, process and content. Participants recommended adding new elements such as the role of companies and stakeholder engagement and the capability of policies to follow innovation. For better sustainability of policies over time and as innovations in technologies increase, the results suggest that governments are willing to commit and invest in policy solutions that include ICTs for older adults who are self-managing, within supportive systems of expert professionals and through amenable collaboration and cooperation in this cross-cutting area. Participants also noted operational and organizational limits of governments as overarching barriers to the implementation of the framework. As a result, several changes (Table 7) were integrated into a revised version of the framework (Fig. 2) which offers a more complete representation of policymaking on older adults’ SMDD using ICTs. Several modifications were made to the definitions of the concepts that are represented in the framework (i.e. policy development and actors), but further investigation is necessary to clarify other definitions (i.e. programs, services, evaluation, external partners and constitutional FPT relations) since the data did not offer that level of detail. The visual representation was also amended to account for recommendations and survey results. Since participants noted that collaboration and cooperation were essential to maintaining sustainable policies over time, this concept was added as cross-cutting all components of policymaking. We have added the concept of stakeholders within the actors section of the framework since participants emphasized that they may be different than partners and could be composed of citizens, patients and companies. The context in which the policies are developed was also said to be highly dependent on the advancements in the clinical and technology sectors. As such, we have added those sectors as two distinct contextual factors. Finally, participants noted that policymaking in the area of older adults’ SMDD using ICTs was highly dependent on operational and organizational factors including human resource capability and financing. As such, they were added as a large bucket around the other concepts of the framework. Overall, the revised framework now portrays the necessary engagements between actors, the collaborative and cooperative nature of processes and influential sectors that need to be considered in policymaking for older adults’ SMDD using ICTs. The revised framework allows for a visual representation of the complex set of systems that interact together in the development of policies, and as such, can guide policymakers in the assessment and prioritization of policy options on the topic of older adults’ SMDD using ICTs. Comparison with prior work.

**Table 7:** Summary of framework changes

Concept	Area within concept	Change
Content		*Requires further investigation
	Programs	*Requires further investigation
	Services	*Requires further investigation
Process	Policy development	The definition of policy development was modified to include a stronger emphasis on the importance of the engagement process
	Evaluation	*Requires further investigation
	Collaboration and cooperation	Collaboration and cooperation are viewed as essential to maintain sustainable policies. As such, it was added to the framework as a cross-cutting activity that occurs throughout the whole process of policymaking
Actors		The definition of actors was modified to include specific wording about actors being embedded within supportive systems and composed of individuals or groups who are experts in the area
	External partners	*Requires further investigation
	Stakeholders	Stakeholders was added to the framework, as it was noted that external and internal partners do not compose the whole realm of stakeholders involved. Stakeholders are diverse and include citizens, patients and companies
Context	Constitutional FPT relations	*Requires further investigation
	Technology (R&D and Industry) sector	The technology sector, including research and development and industry, was noted to be impactful on policymaking and was therefore added to the framework. This sector is one that creates innovations in technology. Policymaking relating to technology would not exist without developments from the technology sector
	Clinical sector	The clinical sector was added to the framework as it is the one that supports the implementation and use of technologies to support patients with their care. Without the clinical sector oversight and support, self-management with technology would not be possible
Operational and organizational factors		Operational and organizational factors are composed of all system-level constraints to policymaking. They include human resource capability and financing

^*^Requires further investigation: level of detail and proposed changes not obtained through survey. Further investigation necessary to identify required changes

The revised framework is unique in that it portrays multiple sectors that intersect in the area of older adults’ SMDD using ICTs. It is composed of the technology sector, the clinical sector and the political system, and considers the roles of various stakeholders and external pressures within all those sectors. It builds on existing approaches to policy analysis such as Walt and Gilson’s model for policy analysis [33], and visually represents the influence of various factors ranging from contextual to organizational.

Our study provides conceptual clarification to the role of governments as a complex system that supports innovation for older adults. In alignment with previous research on policymaking related to digital health innovation in Ontario [20], our study reinforces the need for participatory design approaches via stakeholder engagement at multiple levels during the policymaking process.

Other existing frameworks and models related to the self-management and chronic disease management, such as the Ottawa Charter for Health Promotion [26], Ontario’s Chronic Disease Prevention and Management Framework [27] and the Chronic Care Model [28], provide good foundations for addressing this complex issue, but are limited in providing direction for policymaking specifically. They include public policy as a core function in addressing caring for disease and disability, but do not provide guidance for engaging in it. Our framework provides conceptual clarification for engaging public policy in addressing this issue with the right set of actors, processes and among complex interactions of contextual factors.

### Application of the framework to policymaking

Future work of health policy experts can be supported by the application of this framework, as it offers a foundation upon which to identify all actors and sectors that should compose the consultations. The framework also acts as a reminder for the importance of continuous and engaged cooperation and consultation among all players of the policy environment. In fact, previous research demonstrated the value of cooperation and consultation during policy development and throughout the policy cycle as one that leads to better and more innovative policies that truly address the issue [34]. Finally, the framework can facilitate policymaking as it allows for the identification of numerous policy options that would be generated as the result of consultations and engagements. In policymaking, being able to identify all policy options is critical because it ensures that the most appropriate and most suitable options are selected to address the policy issue [35].

The application of the framework relies on its uptake by policymakers. In this role, policymakers will need to adequately understand the framework and its different components. As mentioned by participants, it is imperative that policymakers develop expertise and knowledge in the technology sector. The need for professional development and training has been highly discussed by the European Commission and the OECD, which have also developed training to support policymakers to make evidence-informed policies and decisions. The European Commission states that policymakers must know ‘how to work with [evidence] and […] integrate it into the complex machinery of policymaking. […] [T]hey need the skills to identify and use relevant evidence in an appropriate way’ [36]. Policymakers will be key in obtaining the breadth of information from all required sectors to be able to answer the complex policy questions. Finally, policymakers’ role will also involve applying the framework to all emerging policy work related to older adults’ SMDD using ICTs.

The framework will be beneficial for policymaking in Ontario, and later in other jurisdictions, as it supports a sustained implementation of ICT-based policies for older adults who self-manage their conditions. This integration comes at a time in which digital health solutions and innovations are part of healthcare and are increasingly playing an important role in care management [14]. The framework will also be beneficial to policy ecosystems as it considers the evolving nature of technological innovation by reminding policymakers of the importance of sustained engagement with key actors in specific sectors. Lastly, the framework conceptually defines policymaking for this complex and modern policy area. It can provide conceptual orientation to the application of change management in the context of policymaking for older adults’ SMDD using ICTs. Broader applicability of the framework to topics outside of policymaking on older adults SMDD using ICTs could also be explored, as there may be similar mutually influencing factors that replicate across multiple topic areas.

## Limitations

This study used a survey design with open-ended questions. While this method is adequate to perform the framework revision exercise, it provided limited accounts of details of the assessments provided by participants. Some limited feedback was obtained through open-ended questions, but do not represent complete and detailed accounts to the extent of qualitative research. Future research that includes semi-structured interviews with policymakers could help gather clarifications on some new components of the framework and on increasing the level of detail for subcategories needing modification.

The survey was administered to managerial- and director-level participants. Its full application in a policy environment would require its use by working-level employees as well. As such, future evaluations of the framework should involve diverse levels of policymaking individuals, including working-level policymakers. In addition, and to supplement the finding that stipulates the need for stakeholder engagement in the policymaking process, future research could look specifically into the role of the advocacy groups, including older adults, into the policymaking process. It may be interesting to determine the frequency and stage of policy development in which they should be involved and to determine whether they feel their voices are heard. It would also be interesting to know more about the groups that are consulted on related policy development inquiries and whether they represent the diversity of perspectives that compose the problematic at stake.

This study had a relatively low response rate (17%) from individuals invited to participate. Our present response rate can be explained by several reported and uncontrollable factors. First, the topic for this study is extremely specific, which inherently decreases the number of potential participants. While we invited many policymakers, it is very likely that many did not qualify or meet the eligibility criteria established in this study. Relatedly and mentioned in the literature, response rate is one metric to quantify the success of online survey, but response representativeness is much more important [37]. Second, during recruitment and data collection, the Government of Ontario had just passed through a provincial election. This may have impacted the availability of management-level employees to participate, as they would have been managing competing priorities such as briefing new elected officials on various policies and programs. Relatedly, COVID-19 remained a high concern during the time of data collection, which for many participants would have been viewed as a more urgent priority. However, a low response rate for this type of topic is not surprising considering the specificity of the topic nor do we view it as having an impact on the validity of the findings due to reasons mentioned above.

## Conclusions

A framework to support policymaking on older adults’ SMDD using ICTs was revised using a survey with policymakers. The results revealed that the provisional framework accurately reflected the realities of policymaking for chronic disease self-management but could benefit from some definitional clarifications and the addition of few additional factors. The framework can be used to encourage research that promotes a common understanding among stakeholders on the legitimacy of various perspectives in policymaking.

The revised framework will be useful for sustained integration of ICT-based interventions within policy initiatives relating to older adults and self-management. The applicability of this framework to jurisdictions outside of Ontario, Canada may require contextual adjustments. Future research should focus on evaluating the implementation of the framework within a policy ecosystem focussed on older adults’ SMDD using ICTs and identifying improvements as needed.

### Supplementary Information

**Additional file 1.** Survey questions.

## Data Availability

The datasets used and/or analyzed during the current study are available from the corresponding author on reasonable request.

## Abbreviations

ICT: Information and communication technology
SMDD: Self-management of disease and disability
AODA: Accessibility for Ontarians with Disabilities Act
FPT: Federal, provincial and territorial
