# Supplementary material for: Framework for policymaking on self-management of health by older adults using technologies
Source: Health Res Policy Syst. 2024 Mar 5;22:32. doi: 10.1186/s12961-024-01119-5 (PMC10913262; doi:10.1186/s12961-024-01119-5)
Supplement: Supplementary file 1 — Additional file 1. Survey questions. [file 12961_2024_1119_MOESM1_ESM.pdf]

Validation Survey - Concept map for policy-making in the area of older adults' self-management disease and disability using ICTs

A) Demographics

1. Please select the ministry or agency for which you work within the Ontario government.

- ◯ Ministry for Seniors and Accessibility
- ◯ Ministry of Health
- ◯ Ministry of Children, Community and Social Services
- ◯ Ministry of Labour, Training and Skills Development
- ◯ Ministry of Economic Development, Job Creation and Trade
- ◯ Other (please specify)

2. What is your role within the Ontario government

- ◯ Policy analyst
- ◯ Team lead
- ◯ Program manager
- ◯ Manager
- ◯ Other (please specify)

3. How many years have you been in your current role?

- ◯ Less than 1 year
- ◯ 1-2 years
- ◯ 2-5 years
- ◯ 5+ years

4. How many years have you been within the Ontario government?

- ◯ Less than 1 year
- ◯ 1-2 years
- ◯ 2-5 years
- ◯ 5-10 years
- ◯ 11-20 years
- ◯ >20 years

Validation Survey - Concept map for policy-making in the area of older adults' self-management disease and disability using ICTs

B) 'The Concept Map' - form and content

In previous steps of the project, some key components were identified as composing current models of policy-making in the area of older adults' self-management of disease and disability (Figure 1 below).

Figure 1: Concept map for policy-making on older adults' self-management of disease and disability using information and communication technologies

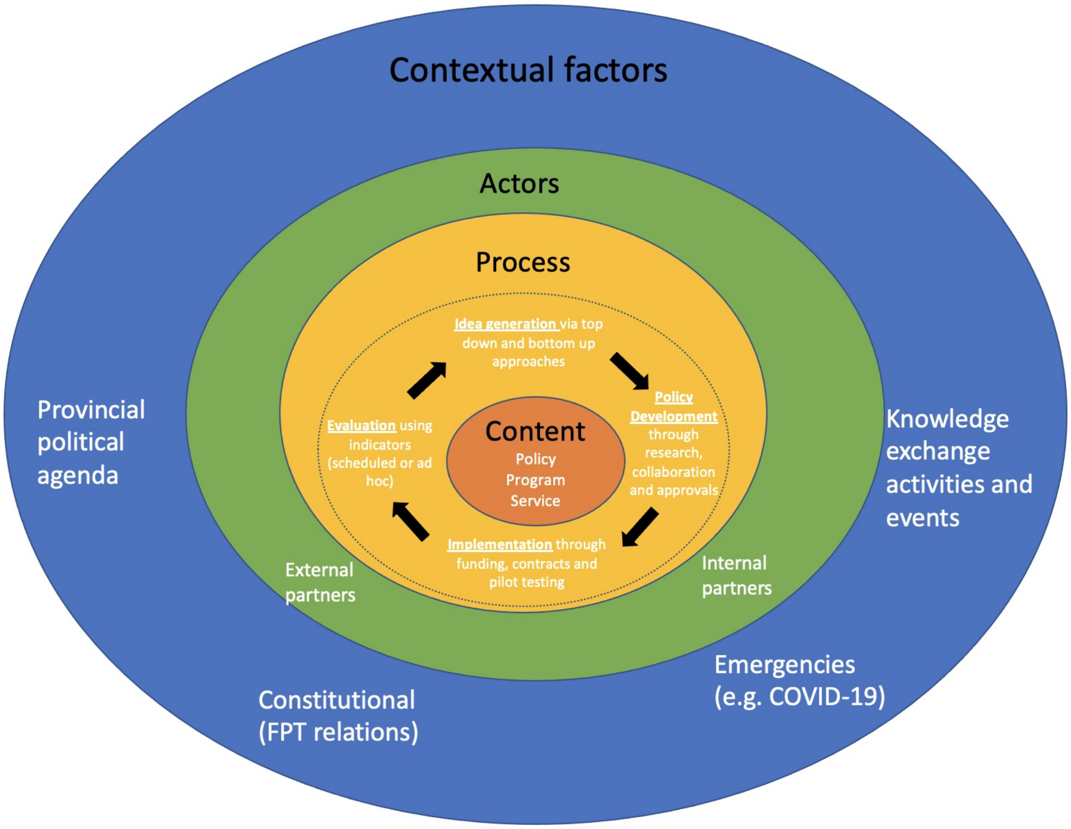

5. Based on the concept map presented in Figure 1, please rate your overall level of understanding and agreement on how well this concept map describes the current and overall policy-making process used by your group on the topic of older adults' self-management of disease and disability using ICTs.

- ◯ Strongly agree
- ◯ Agree
- ◯ Somewhat agree
- ◯ Neither agree nor disagree
- ◯ Somewhat disagree
- ◯ Disagree
- ◯ Strongly disagree

If you disagree to any degree, please use this space to explain why:

6. Would this concept map help you/your group do the following:

	Yes/No
Identify the problem or issue	
Identify an appropriate policy solution	
Identify and describe policy options	
Assess policy options	
Prioritize policy options	
Develop a strategy for furthering adoption of a policy solution	

7. Based on the concept map presented in Figure 1, please rate

whether the definitions **clearly and adequately** explain the concepts related to policy-making for older adults self-management of disease and disability using ICTs.

Choices include:
-Very clear
-Minor modifications required
-Major modifications required
-Not clear at all

	Clarity of definitions
**Contextual factors**<br>External factors that influence stakeholders taking into consideration their origins, processes and content of policies, programs or services.	
**Provincial political agenda**<br>The elected government has the lead on what work needs to occur (based on their electoral platform and promises). The public servants follow the elected governments' lead and decisions. There are strong political orientations.	
**Constitutional FPT relations**<br>There are significant federal priorities that guide or impose upon provincial priorities in Ontario. For example, when funding is involved with transfers from the federal government to the provincial government, Ontario needs to ensure they are meeting the commitments.	
**Emergencies (e.g. COVID-19)**<br>Emergencies refer to external pressures that lead to immediate expansion of current efforts and actions (pivot). It also leads to the development of new and improved supports because of limitations brought by the external pressures. For example, COVID-19 has expanded the use of virtual care in home and community care.	
**Knowledge exchange activities and events**<br>There is often cross-jurisdiction (community, municipal, provincial, federal) analysis to compare and see whether the efforts and actions of other provinces would benefit the province of Ontario. Efforts in other jurisdictions conflict or don't always apply to the province of Ontario, but some components may. The Ontario public service is always on the look-out and learning from other stakeholders, within and across provinces and territories.	
**Actors**<br>All individuals or groups that will contribute in some form to the policy, program or service.	

External partners
Set of partnerships with collaborators that include researchers, foundations, advocacy organizations, community of service providers, businesses, municipalities, and people with lived experience.

Internal partners
Collaboration with other ministries or departments within the Ontario government. This includes intradepartmental and interdepartmental collaboration with groups that have expertise in different areas (policy, program, IT, etc.), working groups, and committees.

Process
Mechanisms that delineate the ideation, creation, implementation and evaluation of policies, programs or services.

Idea generation
The generation of ideas is a hybrid between top down and bottom up approaches whereas ministers or senior management may propose new ideas and avenues to explore or working-level public servants propose new and innovative ways to conduct their work or address a specific issue.

Policy development
Policy development is a complex process that involves research, collaboration between various partners (internal and external) via working groups, advocacy or consultations, and a series of approval levels before a policy is fully shaped and delineated.

Implementation
Policy implementation can take various forms. It can be delivered through funding new programs (or reallocation of funding from previous obsolete programs) where specific amounts are allocated to projects, contracted to external groups to perform the work or deliver a specific program or service, or internally within governemnt departments or agencies. Some implementation may occur through pilot testing where a particular project is tested on a smaller scale before larger implementation.

Evaluation
Evaluations take the form of pre-established performance measurement

and outcomes evaluation where there is a balance between oversight and reporting. Various evaluation instruments and methods are used such as pre-established indicators, metric evaluations, and outcome measurements. Frequency of evaluations differ for each policy where some are more scheduled and others are ad hoc.

Content

Choices in the shape, form and topic of policies, programs or services.

Policy

Existing legislation or regulations relevant to or impacting older adults self-managing diseases or disability using ICTs. For example, the Accessibility for Ontarians with Disabilities Act (AODA) is a law that sets out process for developing and enforcing accessibility standards, and components of that law may be linked to older adults self-managing diseases or disability using ICTs.

Program

Funding program(s) delivered directly by the Government of Ontario or through a community or tier partner, focussing on or impacting older adults self-managing diseases or disability using ICTs.

Service

Direct service to a group that is- or may-serve older adults managing diseases or disability using ICTs.

8. Based on the concept map presented in Figure 1, please rate the **importance** of each concept as it relates to your policy-making on the topic older adults' self-management of disease and disability using ICTs

	Level of importance
Contextual factors	
Provincial political agenda	
Constitutional FPT relations	
Emergencies (e.g. COVID-19)	
Knowledge exchange activities and events	
Actors	
External partners (e.g. researchers, foundations, advocacy organizations, services providers, businesses, municipalities or people with lived experience)	
Internal partners (e.g. other ministries, departments , teams or units within the government of Ontario)	
Process	
Idea generation	
Policy development	
Implementation	
Evaluation	
Content	
Policy	
Program	
Service	

Choices include:
-Extremely important
-Very important
-Moderately important
-Neutral
-Slightly important
-Low importance
-Not improtant at all

Please rate your agreement with the statements below about the concept map presented in Figure 1 and include comments if you disagree:

9. I find the concept map visually appealing

- ◯ Strongly agree
- ◯ Agree
- ◯ Somewhat agree
- ◯ Neither agree nor disagree
- ◯ Somewhat disagree
- ◯ Disagree
- ◯ Strongly disagree

Comments

10. The format of the concept map makes it easy to understand

- ◯ Strongly agree
- ◯ Agree
- ◯ Somewhat agree
- ◯ Neither agree nor disagree
- ◯ Somewhat disagree
- ◯ Disagree
- ◯ Strongly disagree

Comments

11. The concept map is usable in its current form

- ◯ Strongly agree
- ◯ Agree
- ◯ Somewhat agree
- ◯ Neither agree nor disagree
- ◯ Somewhat disagree
- ◯ Disagree
- ◯ Strongly disagree

Comments

12. List any additional factors that you think are important when developing new or modernizing policy on older adults' self-management of disease and disability using ICTs, that may not be represented in the concept map depicted in Figure 1. For example, are there important policy issues for which this concept map may not be suitable?

13. How can innovations in technology to support older adults to self-manage be easily integrated in Government of Ontario policies, programs and services through time? List any ideas in terms of context, actors, processes, content or other that would need to be considered for better integration of technological innovations that support older adults' self-management into existing or new policies.

Context

Actors

Process

Content

Other

14. Thinking about technology and digitalization of healthcare, what do you think would allow policies within the Government of Ontario to be sustainable over time?

15. Please provide any additional comments about the concept map's form and content:

Validation Survey - Concept map for policy-making in the area of older adults' self-management disease and disability using ICTs

C) 'Setting' – People and organization as implementers

Figure 1: Concept map for policy-making on older adults' self-management of disease and disability using information and communication technologies

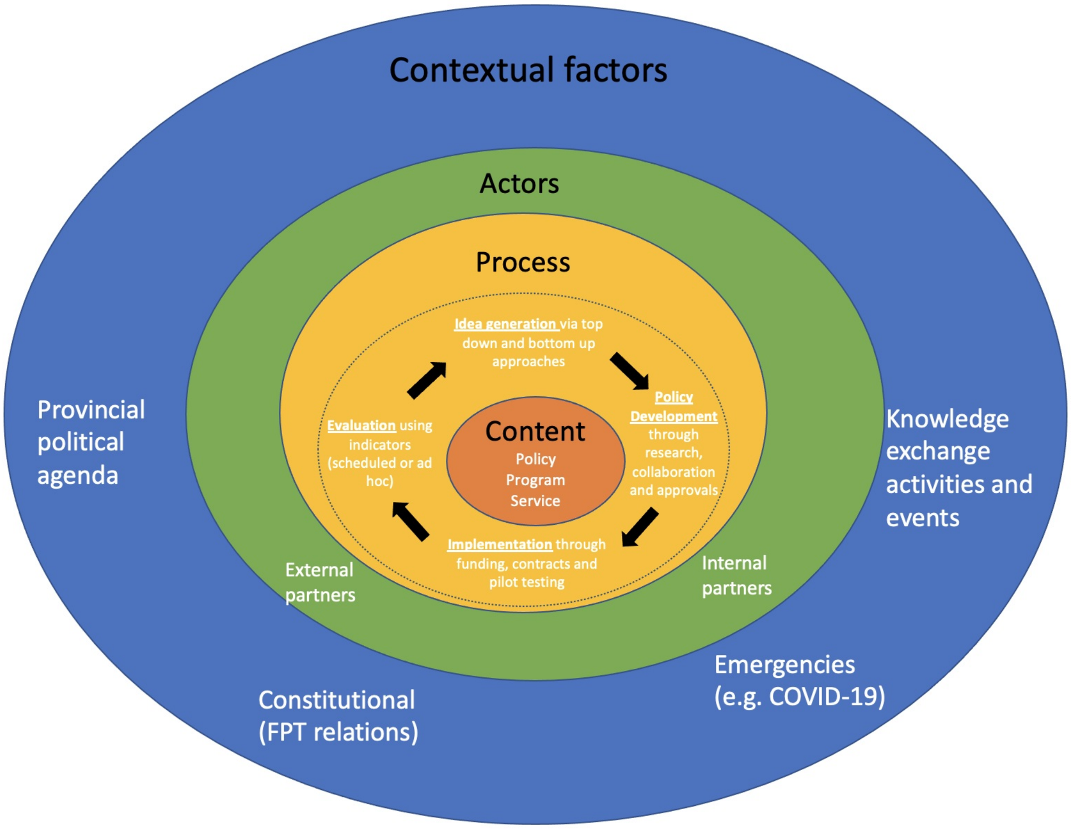

16. Would you say that you could be a user or strong proponent of the concept map in your policy-related tasks specific to older adults' self-management of disease or disability using ICTs?

- ◯ Definitely would
- ◯ Probably would
- ◯ Possibly
- ◯ Probably would not
- ◯ Definitely would not

17. Who would be best positioned to use and implement this concept map to guide their policy work? Please select all that apply.

- ☐ Policy analysts
- ☐ Team leads
- ☐ Program managers
- ☐ Managers
- ☐ Directors
- ☐ Other (please specify)

Please rate your agreement with the statements below:

18. I have the necessary expertise to **understand** such a concept map in my policy-related activities

- ◯ Strongly agree
- ◯ Agree
- ◯ Somewhat agree
- ◯ Neither agree nor disagree
- ◯ Somewhat disagree
- ◯ Disagree
- ◯ Strongly disagree

19. I have the necessary expertise to **implement** such a concept map in my policy-related activities

- ◯ Strongly agree
- ◯ Agree
- ◯ Somewhat agree
- ◯ Neither agree nor disagree
- ◯ Somewhat disagree
- ◯ Disagree
- ◯ Strongly disagree

20. I see the value of a concept map to guide policy-making in the area of older adults' self-management of disease and disability using ICTs.

- ◯ Strongly agree
- ◯ Agree
- ◯ Somewhat agree
- ◯ Neither agree nor disagree
- ◯ Somewhat disagree
- ◯ Disagree
- ◯ Strongly disagree

21. I view this concept map as sustainable over time

- ◯ Strongly agree
- ◯ Agree
- ◯ Somewhat agree
- ◯ Neither agree nor disagree
- ◯ Somewhat disagree
- ◯ Disagree
- ◯ Strongly disagree

22. This concept map enables a continuous monitoring and integration of innovation in policy-making (i.e. new technological developments to support self-management)

- ◯ Strongly agree
- ◯ Agree
- ◯ Somewhat agree
- ◯ Neither agree nor disagree
- ◯ Somewhat disagree
- ◯ Disagree
- ◯ Strongly disagree

23. The Government of Ontario and my organization could benefit from a concept map or framework to guide policy-making on the topic of older adults' self-management of disease and disability using ICTs.

- ◯ Strongly agree
- ◯ Agree
- ◯ Somewhat agree
- ◯ Neither agree nor disagree
- ◯ Somewhat disagree
- ◯ Disagree
- ◯ Strongly disagree

24. The Government of Ontario and my organization are ready to implement a concept map or framework, like the one proposed, to guide policy-making on the topic of older adults' self-management of disease and disability using ICTs.

- ◯ Strongly agree
- ◯ Agree
- ◯ Somewhat agree
- ◯ Neither agree nor disagree
- ◯ Somewhat disagree
- ◯ Disagree
- ◯ Strongly disagree

25. The Government of Ontario and my organization have the necessary human and financial resources to implement this concept map

- ◯ Strongly agree
- ◯ Agree
- ◯ Somewhat agree
- ◯ Neither agree nor disagree
- ◯ Somewhat disagree
- ◯ Disagree
- ◯ Strongly disagree

26. Please provide any additional comments about the people and organizations (Government of Ontario) as implementers of the concept map:

Université d'Ottawa | University of Ottawa

Validation Survey - Concept map for policy-making in the area of older adults' self-management disease and disability using ICTs

D) Additional thoughts and comments

27. Please provide any additional comments on the topic of policy-making for older adults who are self-managing their disease or disability using ICTs.
